# Impact of Enhanced Outreach Activities on Immunization Coverage in Pakistan: Evidence from Secondary Data Analysis (2011–2021)

**DOI:** 10.3390/vaccines14070636

**Published:** 2026-07-20

**Authors:** Shah Nawaz Jiskani, Musa Khan, Mohammed Osama Mere, Wei Xia, Atta Ur Rehman

**Affiliations:** 1 WHO Country Office, Prime Minister’s National Health Complex, Park Road, Chak Shahzad, Islamabad 44000, Pakistan; jiskanis@who.int (S.N.J.); xiaw@who.int (W.X.); attaurrehman1987@yahoo.com (A.U.R.); 2 Federal Directorate of Immunization (FDI), Prime Minister’s National Health Complex, Park Road, Chak Shahzad, Islamabad 44000, Pakistan; 3 World Health Organization, Regional Office for the Eastern Mediterranean, Monazamet El Seha El Alamia Street, Extension of Abdel Razak El Sanhouri Street, P.O. Box 7608, Nasr City, Cairo 11371, Egypt

**Keywords:** expanded programme on immunization, routine immunization, enhanced outreach activities, immunization coverage, immunization agenda 2030

## Abstract

Background: Despite noteworthy progress in the Expanded Program on Immunization (EPI) for improving vaccination coverage worldwide, inequalities in vaccine uptake and accessibility persist in several middle- and low-income states. Pakistan’s routine vaccination uptake varies greatly between federal geographical regions and continues to fall below the standard threshold needed to attain optimal herd immunity. The Federal Directorate of Immunization (FDI) in collaboration with the provincial Expanded Program on Immunizations implemented Enhanced Outreach Activities (EOAs) to boost immunization delivery in selected districts in order to address immunization coverage gaps in marginalized and difficult-to-reach communities. The purpose of this secondary data research was to evaluate whether EOAs improved immunization coverage in the country. Methods: The Federal Directorate of Immunization’s longitudinal panel analysis of district–month administrative data for the years 2011–2021 have been analyzed. The research investigation reviewed 158 districts’ monthly vaccination records for multiple antigens, covering regions where EOAs were successfully carried out in particular months. National and subnational analyses were carried out to measure geographical variations in the impact of Enhanced Outreach Activities (EOAs). The average vaccination coverage during EOAs and non-EOAs months was compared using the paired *t*-test. Results: An aggregate of 2430 months with EOAs were included in the analysis of 19,068 monthly district observations. National level analysis revealed higher average vaccinations during the EOA months for all antigens, but a statistically significant increase was observed for IPV and measles second dose (*p* < 0.05) only. Provincial-level results indicate that the impact was more evident in underserved regions. Khyber Pakhtunkhwa, Balochistan, Punjab, and Sindh demonstrated statistically significant increases in vaccination counts for most antigens. Due to variations in baseline population coverage, as well as health care sector capability, the extent of improvements differed throughout regions. Conclusions: In Pakistan, routine immunization counts has been improved via enhanced outreach initiatives, especially in provinces with lower initial vaccination rates and restricted accessibility to services. Strengthening outreach strategies, together with consistent fixed-site immunization services, is likely to accelerate progress toward equitable immunization coverage and contribute to achieving the Immunization Agenda 2030 goals.

## 1. Introduction

Immunization has been recognized as one of the most cost-effective interventions to reduce morbidity and mortality in children. The international institutionalization of immunization began in 1974 as the WHO (World Health Organization) established the EPI (Expanded Program on Immunization) after the effective eradication of smallpox [1,2,3]. Following this, immunization programs achieved significant gains in VPDs (vaccine-preventable diseases) worldwide and remain instrumental for improving children’s survival in low-income and middle-income nations [4]. The national Expanded Program on Immunization in Pakistan was started in 1978 to immunize children against the major VPDs (vaccine-preventable diseases). The program expanded over time with additional antigens, consistent with international recommendations and epidemiological trends. The recent vaccination schedule aimed to protect children against 12 vaccine-preventable diseases and required six health care system contacts during the initial 15 months to achieve adequate immunity [5,6]. Immunization rates remain suboptimal in federal areas and provinces despite the continuing EPI initiatives in the country. GVAP (Global Vaccine Action Plan 2011–2020) established the minimum national level targets of 90% and district level targets of 80% immunization coverage, measuring DTP-3 coverage as the EPI performance indicator [7,8]. Nonetheless, the findings of the national and third-party surveys showed variations in vaccination coverage among provinces and federal areas [6].

Pakistan continues to be one of the nations with a high number of zero-dose and under-vaccinated children. Incomplete immunization is due to a number of factors such as socioeconomic disparities, inadequate health services access, parental reluctance, and logistical challenges within service delivery systems [9]. These challenges are especially noticeable in isolated remote regions, along with peri-urban settlements, where utilization of fixed medical services remains limited. The country offers regular childhood vaccinations through fixed medical centers, outreach initiatives, and mobile vaccination staff [10]. Fixed-location networks may not adequately reach marginalized communities, even when they offer organized delivery of regular vaccinations. Assessments of health care facilities have shown that routine vaccination administration has been affected by operational constraints along with variations in the quality of service [11]. Worldwide, outreach activities and mobile vaccination strategies remain vital in hard-to-reach areas to provide equitable access to EPI services [12,13]. EPI in Pakistan has been implemented. Enhanced Outreach Activities (EOAs) are a set of systematic, intensified interventions designed to extend immunization services beyond routine facility-based delivery to reach children who are often missed. EOAs are routine outreach activities that are periodically intensified through the provision of adequate operational resources, particularly POL, for transportation, along with strengthened supervision and monitoring—components often limited in standard outreach due to funding constraints. Through this approach, WHO provided additional support to supervisors and vaccinators to expand coverage of routine immunization services in underserved, remote, and hard-to-reach areas. EOAs are informed by global best practices, including the WHO’s Reaching Every District (RED) framework, and typically encompass several interrelated components. Microplanning uses local data to map target populations, identify underserved and defaulting children, schedule outreach sessions, and plan logistics and resources, ensuring a data-driven approach to coverage and equity in EOA months. EOA month was a district-month with a round of EOAs lasting 3–12 days, and these EOAs were implemented in selected union councils with low coverage. Social mobilization engages communities through health education, communication, and dialog with caregivers and local leaders to build awareness, trust, and demand for immunization services. Supportive supervision and monitoring involve structured supervisory visits, feedback, and problem-solving support to strengthen frontline performance, reinforce data use, and ensure quality service delivery. Additionally, targeted outreach strategies, such as mobile teams, periodic intensification campaigns, and integrated service delivery at outreach sites, aim to improve access in geographically or socioeconomically hard-to-reach areas. Catch-up vaccination is inherently part of the EOA strategy, contributing to the observed increases. Collectively, EOAs are implemented to reduce inequities in immunization coverage by systematically identifying and reaching unserved and under-vaccinated children, strengthening community participation, and enhancing health system accountability and responsiveness.

Targeting zero-dose children, increasing primary medical care networks, and resolving disparities in vaccination coverage all remain priorities included in the Immunization Agenda 2030 on an international level [14]. Additionally, immunization continues to be essential for reaching Sustainable Development Goal 3, especially in terms of lowering mortality rates among children under five while increasing universal provision of basic medical care [15]. Despite the fact that EOAs have long been implemented in Pakistan, there continues to be a dearth of empirical data assessing their quantifiable influence on immunization coverage. Thus, the purpose of this secondary data analysis is to evaluate how EOAs impact vaccination coverage in Pakistan in order to gather evidence that will guide the development of strategies towards enhancing equitable immunization delivery.

## 2. Methodology

This survey applied a longitudinal panel analysis of district-month administrative data to assess the impact of Enhanced Outreach Activities on immunization coverage in the country. A monthly record of 11 years (January 2011–December 2021) was scrutinized. Federal Directorate of Immunization (FDI) and Provincial Expanded Program on Immunization (EPI) collected statistics through the district, provincial, and national reportage system, which was analyzed. The information set comprised data on the provinces, districts, years, months, EOA status (applied or not applied), and total children immunized for every antigen. An EOA month was defined as any district-month in which at least one round of Enhanced Outreach Activities was implemented in one or more union councils within the district. EOAs were conducted in selected union councils for 3–12 days per round, and district-level exposure was assigned based on the occurrence of any EOA activity within the district during a given month. The analysis compared the immunization outcome in the districts with reference to EOAs. Immunization data statistics were compiled for BCG Vaccine (Bacillus Calmette–Guérin), OPV (Oral Polio Vaccine), Pentavalent vaccine, PCV (Pneumococcal Conjugate Vaccine), Rotavirus vaccine, IPV (Inactivated Polio Vaccine) and MR Vaccine (Measles Rubella Vaccine). PCV was introduced in a phased manner during 2014–2016, IPV in 2015, and rotavirus vaccine in 2017; analyses for each antigen were restricted to the post-introduction period, with pre-introduction months excluded. Inclusion criteria: prerequisite districts with reliable, consistent and complete data records in the selected period. The use of enhanced outreach initiatives was the main exposure predictor. EOA eligibility was determined using program historical documents at the district-by-month levels.

The total number of children that were vaccinated with each antigen in the district area was used to quantify changes in regular vaccination performance, which was the main outcome of concern. Considering that pentavalent-III uptake represents the completion of the primary vaccine series, it was considered a crucial sign of the effectiveness of the everyday immunization program. In light of their program significance as well as their function in tracking coverage along with service utilization patterns, BCG rates and the first dose of MR were likewise analyzed. The analysis sought to evaluate coverage changes related to the EOAs months while minimizing (not considering) the effect of constant district-level cofactors such as baseline availability of services, health care infrastructure, and demographic profile of the children. Before analysis, the data was cleaned, checked for accuracy, and arranged by region and duration. Overall trends in specific antigen-based vaccination effectiveness over the course of time have been presented using frequencies as well as percentages. The *t*-test for paired samples was used to evaluate the impact of EOAs on immunization outcomes. As immunization outcomes were compared over two distinct periods among the same districts, the aforementioned statistical approach was chosen. The assessment analyzed the statistical significance of the average variation in vaccination effectiveness during EOA intervals by examining pairs of data from the same administrative areas. This method allowed the investigation to concentrate on temporal shifts related to EOA administration while taking into account constant context-specific factors across regions. Before doing the inferential evaluation, the pairing *t*-test’s basic presumptions, such as the variance in scores about the normal distribution, were considered. *p*-values beneath the probability threshold of 0.05 were taken as proof of statistically significant variations in immunization performance. Impact assessments were carried out at the national, provincial, and town levels to investigate differences in effects throughout administrative divisions. The percentage of districts showing statistically noteworthy improvement was ascertained by calculating the average variations for each antigen. The assessment of the overall impact, along with the geographical consistency of EOA-associated outcomes, was made achievable by this multiple-level analysis approach.

Shaheed Zulfiqar Ali Bhutto Medical University’s ASRB (Advanced Studies & Research Board) and IRB (Institutional Review Board) both granted ethical authorization for the project. The Federal Directorate of Immunization permitted the utilization of routine vaccination records officially. Confidentiality and adherence to ethical norms were ensured in the information being used in analysis, which only comprised aggregated district-specific information with no personal identification.

## 3. Results

The findings are presented as an analysis of Enhanced Outreach Activities utilizing administrative level data (2011 to 2021), contextualized within the trends in immunization coverage at the national, province, and district levels.

### 3.1. National Overview of Enhanced Outreach Activities

Out of the 158 total districts countrywide at that time, 148 applied one round (at least) of the EOAs during the survey period, as presented in Table 1. District-wise microplans that targeted specific underprivileged areas were used to carry out EOAs. There were 19,068 district calendar month observations during the 11 years for analysis. Of them, 2430 district-months represented EOAs months, whereas 16,638 district-months represented non-EOAs months.

#### 3.1.1. Impact of EOAs at the National Level

Average immunization counts for the majority of antigens were higher during EOA periods in comparison to non-EOA periods at the country level. IPV (Inactivated Polio Vaccine, *p* = 0.02) and Measles II (*p* < 0.00) were observed to be significant for two of the antigens. Even though the average levels of 11 out of 16 antigens were higher throughout EOA months, these variation aggregates were not statistically significant at the national level, as shown in Table 2.

#### 3.1.2. Impact of EOAs at the Provincial Level

EOAs were applied in each of the ten districts of the AJK (Azad Jammu and Kashmir); nevertheless, no statistically significant rise for any antigen was seen during EOA months, and there was no substantial significance between EOA and non-EOA months. EOAs were carried out in 33 areas in Balochistan, and all of the 16 antigens showed a statistically significant increase (*p* < 0.001). There was a significant correlation between EOAs and better vaccination uptake, as seen by the significantly higher monthly average vaccination numbers per district throughout EOA months. In GB (Gilgit Baltistan), EOAs were carried out in all the 10 districts, whereas immunization rates were typically greater throughout the EOA months, six antigens (OPV-0, PCV2 and 3, Rota1 and 2, and IPV) indicated statistically noteworthy increases, whereas the remaining antigens demonstrated non-significant improvements. EOAs were introduced within both the administration districts of the Islamabad Federal Territory, and during EOA months, there were notable increases for each antigen, reflecting better service usage. EOAs were carried out in 26 districts in the Khyber Pakhtunkhwa, and all 16 antigens showed substantial increases (*p* < 0.001). In the same way, all antigens exhibited notable increases throughout the seven tribal regions that carried out roughly 16 rounds for EOAs. EOAs were implemented throughout the 36 districts of Punjab, although the quantity of sessions varied. When comparing EOA months to non-EOA months, vaccination frequencies were considerably higher across all antigens. Similarly, EOAs were carried out in every district in Sindh (Karachi being documented as one administrative entity). Significant improvements were noted for every antigen, suggesting a uniform positive outcome throughout the province, as shown in Table 3.

#### 3.1.3. Impact Significance at the Districts-Level

Among the 148-districts observed, IPV had the largest percentage of regions with notable vaccination improvements (83%), promptly followed by OPV0 (80%), as well as Measles2 (79%). Penta3 (73%) and BCG (70%) demonstrated moderate gains, whereas Rota1 had the lowest percentage of districts with notable increases (55%). All things considered, these results indicate that EOAs were most often linked to improvements in IPV, OPV0, and Measles2, while the effect was relatively smaller for Rota1, as shown in Table 4.

### 3.2. Immunization Coverage Trends Across Pakistan (PDHS 2018 vs. TPVICS2022)

Fully immunized children’s coverage was 66% in 2018 according to the Pakistan Demographic and Health Survey. Substantial disparities were evident in terms of provinces and federating area-wise comparison. Fully immunized children’s coverage was 75% in AJK, 80% in Punjab, 68% in Islamabad, 57% in GB, 49% in Sindh, 55% in KP, 30% in KPMD, and 29% in Balochistan. Data from the Third-Party Verification Immunization Coverage Survey (TPVICS) of 2022 was also compared to assess the fully immunized coverage among children in Pakistan. Fully immunized children’s coverage was 90% in AJK, 89% in Punjab, 81% in Islamabad, 71% in GB, 69% in Sindh, 61% in KP, 41% in KPMD, and 37% in Balochistan.

**Figure 1:**
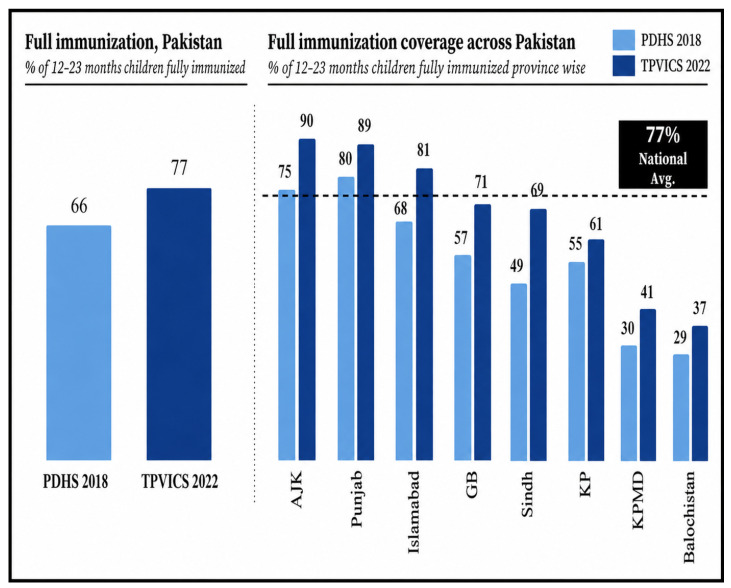
Full Immunization Children’s Coverage across Pakistan (PDHS 2018 vs. TPVICS 2022).

Data from Full Immunization Children’s Coverage across Pakistan (PDHS 2018 vs. TPVICS2022) showed significant differences in immunization coverage in different areas in Pakistan, as presented in Figure 1. Percentage analysis from PDHS 2018 and TPVICS 2022 requires measures to enhance immunization coverage among Children in Pakistan.

**Figure 2:**
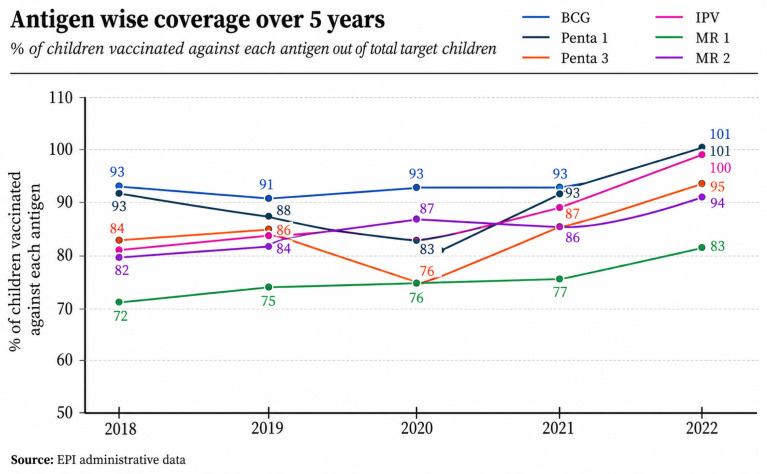
Routine Immunization Coverage for all Antigens over 5 years.

The immunization coverage data from the last five years showed a significant improvement in the immunization coverage for BCG, IPV, Penta I and, III, and Measles-Rubella I and II, as shown in Figure 2.

## 4. Discussion

Ensuring equitable accessibility to vaccinations continues to be a top concern for international public health. Since the Expanded Program on Immunization (EPI) was established in 1974, vaccination initiatives have historically decreased the morbidity and mortality from VPDs (vaccine-preventable diseases) during the previous 50 years. In spite of substantial improvement in expanding immunization coverage worldwide, inequalities in vaccine accessibility and vaccine uptake continue to impact several low-income and middle-income states. These inequalities were ever so often linked with geographic remoteness, socioeconomically deprived population cohorts, health care system restrictions, and vaccination service delivery barriers [16,17,18]. Strengthening vaccine delivery systems and putting focused initiatives in place to reach marginalized communities will be required to address such discrepancies.

Increasing routine vaccination coverage continues to be an important EPI priority in Pakistan. Despite aggregate coverage continuing to lie below the 95% benchmark advised by the World Health Organization for attaining optimal herd immunity, national survey statistics show gains in the percentage of completely vaccinated children in the last few years [19,20]. Some districts, as well as provinces, have consistently lower coverage, which suggests that a significant proportion of children remain either not vaccinated at all or just partially vaccinated. These missed opportunity gaps raise the possibility of VPD (vaccine-preventable disease) outbreaks and emphasize the need for improved immunization service delivery methods. Significant differences still exist between districts and provincial governments despite progress in national coverage. A lower acceptance of vaccinations given subsequently in the immunization schedule indicates difficulties in guaranteeing the vaccination schedule is completed. Inadequate caregiver knowledge of vaccination timelines, an inadequate number of qualified vaccinators, poor health care infrastructure, and restricted access to medical facilities in impoverished and remote regions all constitute contributing determinants. Parental consent to routine immunizations has also been found to be impacted by vaccine hesitancy and disinformation [21,22,23].

The current research investigation used administrative district-wise data collected over an eleven-year period to evaluate the impact of Enhanced Outreach Activities (EOAs) on the regular immunization outcomes. A number of antigens, including IPV and the second dose of the measles vaccination, showed statistically significant gains at the national level. Though an increase in immunization counts was noticed for some other antigens throughout the EOA’s month, a statistically significant impact was not reliably accomplished across all the antigens. These results revealed that while outreach activities can enhance the vaccine uptake, their impact as measurable may fluctuate depending on the implementation intensity, number of days, populace characteristics, and local immunization delivery system capacity. Statistical significance was assessed using *p*-values to determine whether observed changes were unlikely to have occurred by chance, while practical significance was interpreted in terms of the magnitude and programmatic relevance of changes in vaccine delivery. This distinction ensured that results were not only statistically valid but also meaningful for immunization program performance and public health decision-making.

Analysis at the provincial level showed significant heterogeneity in the EOAs effectiveness. In Balochistan, EOAs were linked with statistically significant intensifications across all the antigens. This result remains particularly imperative given that the province has historically stated some of the lowest vaccination coverage levels in the country due to the geographical barriers, dispersed populaces, and limited health care infrastructure. Outreach approaches therefore seem to play an important role in extending immunization services to societies that are not adequately served by the fixed health centers.

Similarly, provinces such as Khyber Pakhtunkhwa (KP) and the prior tribal districts proved significant progress in immunization uptake during the EOA months. These regions are categorized by the difficult geographical terrain, security-linked challenges, and factually underserved inhabitants. The outcomes therefore recommend that outreach-based initiatives may generate better relative improvements in situations where accessibility barriers remain noticeable.

In Islamabad, statistically significant rises were observed across all the antigens during the EOA months, demonstrating the potential effectiveness of the structured outreach initiatives when supported by adequate administration capacity and supportive supervision. In contrast, Azad Jammu and Kashmir did not demonstrate statistically significant progress in spite of the EOAs’ months, possibly reflecting higher baseline vaccination coverage and fewer outreach rounds.

Punjab and Sindh provinces also presented statistically significant increases across most of the antigens during the EOA months, although the magnitude of the improvement varied across districts. These results suggest that the effectiveness of outreach activities may depend on the quality of the microplanning, communal mobilization strategies, and the local health system capacity.

The impact of EOAs in underserved areas observed in this survey aligns with the international evidence representing the effectiveness of outreach-based immunization strategies in improving vaccination coverage among hard-to-reach populations [24,25,26]. Studies in several low-income and middle-income countries have revealed that integrated outreach strategies, mobile vaccination teams, and community-based vaccination programs can significantly increase vaccine uptake by reducing geographic and socioeconomic barriers to access. For instance, integrated periodic outreach programs applied in Ethiopia have positively expanded vaccination coverage in remote regions by delivering services directly within communities [27]. Similarly, surveys from Uganda have demonstrated that outreach immunization services improve access to vaccination among rural populations when combined with community mobilization and awareness campaigns [28]. Evidence from Kenya has also shown that reminder systems and community engagement strategies can significantly improve vaccination timeliness and completion rates [29].

Global analyses focusing on zero-dose children further emphasize the importance of targeted outreach interventions in improving equity in immunization coverage. Mapping studies indicate that children who have not received any routine vaccines are often concentrated in areas characterized by poverty, limited health infrastructure, and geographic isolation [30]. Overall, the findings of this study indicate that Enhanced Outreach Activities contribute positively to routine immunization performance in Pakistan, particularly in provinces with historically low coverage and structural access barriers. However, outreach strategies alone may not be sufficient to achieve universal immunization coverage. Strengthening fixed-site immunization services, ensuring reliable vaccine supply chains, improving workforce capacity, enhancing caregiver awareness, and addressing vaccine hesitancy remain essential complementary strategies.

From a policy perspective, these findings align with the priorities of the Immunization Agenda 2030 (IA2030), which emphasizes equitable access to vaccines, reaching zero-dose children, and strengthening primary health care systems. Improving immunization coverage also contributes directly to Sustainable Development Goal 3 by reducing under-five mortality and preventing outbreaks of vaccine-preventable diseases. Continued investment in outreach programs, strengthened monitoring systems, and adaptive microplanning approaches may therefore play an important role in accelerating progress toward national and global immunization targets.

## 5. Conclusions

This study evaluated the impact of Enhanced Outreach Activities on routine immunization performance in Pakistan using district-level administrative data over an eleven-year period. The findings demonstrate that EOAs contributed to improvements in vaccination uptake, particularly in provinces characterized by lower baseline coverage and structural barriers to health service access. Significant increases in vaccination counts during EOA months were observed in several provinces, suggesting that outreach-based strategies can play an important role in extending immunization services to underserved populations.

The observed variability in impact across provinces highlights the importance of contextual factors such as health system capacity, geographic accessibility, and the quality of microplanning and community engagement efforts. While outreach activities appear to be effective in improving access to immunization services in hard-to-reach areas, they should be implemented as part of a broader strategy that includes strengthening fixed-site services, improving vaccine supply chains, and addressing demand-side barriers such as caregiver awareness and vaccine hesitancy.

From a policy perspective, Enhanced Outreach Activities represent a valuable operational approach for advancing equitable immunization coverage and reaching children who remain outside routine services. Strengthening and scaling such outreach strategies could contribute to achieving the goals of the Immunization Agenda 2030 and Sustainable Development Goal 3 by improving vaccine equity and reducing preventable childhood morbidity and mortality. Continued investment in outreach interventions, combined with strengthened monitoring and adaptive program planning, will be essential to accelerate progress toward universal immunization coverage in Pakistan.

## 6. Strengths and Limitations

This study provides a comprehensive assessment of the impact of Enhanced Outreach Activities (EOAs) on routine immunization in Pakistan using district-level administrative data collected over an eleven-year period (2011–2021). A major strength of the study is the use of a large national dataset covering 148 districts, which enabled comparison of vaccination trends across provinces with diverse geographic and health system contexts. The use of statistical analysis to compare vaccination uptake during EOA and non-EOA months further allowed an objective assessment of the potential contribution of outreach activities to routine immunization performance. In addition, the long study period provides valuable insights into variations in program implementation over time and offers relevant evidence for policymakers and immunization program managers.

The study has several limitations that should be considered while interpreting the findings. First, the analysis was conducted as a descriptive programmatic assessment using routinely collected administrative immunization data and was not designed as a longitudinal econometric or causal inference study. Therefore, the analytical approach focused on descriptive trend analysis and paired comparisons across predefined program periods rather than advanced district-level panel regression models with fixed effects or count-data specifications. Second, the study relies on observational administrative data, which may be affected by reporting errors, inconsistencies, and incomplete records. Although denominators from microplans were used, district-level variations and other unmeasured contextual factors could have influenced the results. Seasonal variation, geographic accessibility, population movement, and other concurrent programmatic activities were not controlled for and may have contributed to the observed differences between EOA and non-EOA periods. Third, the use of aggregated district-level data limited the ability to examine individual-level determinants of vaccination uptake, including socioeconomic status, caregiver awareness, health-seeking behavior, and other household-level factors. In addition, variations in the intensity, implementation quality, and operational effectiveness of EOAs across districts and time periods may also have influenced the observed findings. Finally, because the study was conducted within the scope of an approved descriptive programmatic analysis framework and existing data-use permissions, additional analytical restructuring beyond the original protocol was outside the scope of the present study. Furthermore, the observational design does not permit definitive causal inference regarding the direct impact of EOAs on immunization coverage. Despite these limitations, the study provides important programmatic evidence on the potential contribution of outreach-based strategies in strengthening immunization services and improving vaccination performance in Pakistan.

## Figures and Tables

**Table 1:** Selected EOA Districts at the National Level.

Province	Total Districts	EOA Districts
AJK	10	10
Balochistan	35	33
GB	10	10
Islamabad	2	2
KP	27	26
KPMD	8	7
Punjab	36	36
Sindh	30	24
National	158	148

**Table 2:** Comparison of Vaccination Intake in EOAs vs. non-EOAs months at the National Level.

Antigen	Non-EOA Month Mean Number of Vaccinations per Month per District	EOA Month Mean Number of Vaccinations per Month per District	*p* Value	Statistically Significant Increase
BCG	3681.83	3815.20	0.25	No
OPV0	2990.20	3131.80	0.18	No
OPV1	3491.74	3593.18	0.34	No
OPV2	3275.71	3352.50	0.44	No
OPV3	3199.86	3287.97	0.37	No
Penta1	3464.93	3595.93	0.22	No
Penta2	3248.37	3356.73	0.28	No
Penta3	3174.99	3294.16	0.22	No
PCV1	3743.99	3587.84	0.15	No
PCV2	3521.59	3347.23	0.09	No
PCV3	3433.05	3284.91	0.14	No
Rota1	3817.99	3553.53	0.13	No
Rota2	3549.82	3343.67	0.07	No
IPV	3134.81	3378.62	0.02	Yes
Measles1	3232.17	3385.55	0.13	No
Measles2	2617.94	2915.20	0.00	Yes

**Table 3:** Antigen-wise comparison of Vaccination Intake in EOA Months vs. Non-EOA Months.

Antigen	Non-EOA Month Mean Number of Vaccinations per Month per District	EOA Months Mean Number of Vaccinations per Month per District	*p* Value	Statistically Significant Increase	Non-EOA Month Mean Number of Vaccinations per Month per District	EOA Month Mean Number of Vaccinations per Month per District	*p* Value	Statistically Significant Increase	Non-EOA Month Mean Number of Vaccinations per Month per District	EOA Month Mean Number of Vaccinations per Month per District	*p* Value	Statistically Significant Increase	Non-EOA Month Mean Number of Vaccinations per Month per District	EOA Month Mean Number of Vaccinations per Month per District	*p* Value	Statistically Significant Increase
	Azad Jammu and Kashmir				Balochistan				Gilgit Baltistan				Islamabad			
*BCG*	1177.35	1066.70	0.40	No	679.82	1057.93	0.00	Yes	372.31	379.98	0.78	No	2205.64	2915.37	0.00	Yes
*OPV0*	934.61	983.95	0.67	No	255.28	450.16	0.00	Yes	213.16	244.74	0.05	Yes	1921.66	2737.47	0.00	Yes
*OPV1*	1136.58	1102.95	0.79	No	648.91	1062.15	0.00	Yes	368.60	388.43	0.47	No	1884.21	2232.29	0.00	Yes
*OPV2*	1115.10	1145.15	0.81	No	564.12	927.54	0.00	Yes	338.65	354.63	0.47	No	1787.58	2072.90	0.00	Yes
*OPV3*	1083.84	1131.85	0.70	No	517.35	867.29	0.00	Yes	330.92	346.81	0.46	No	1706.51	1928.61	0.00	Yes
*Penta1*	1136.92	1127.00	0.94	No	649.70	1060.18	0.00	Yes	368.96	386.36	0.53	No	1884.68	2262.24	0.00	Yes
*Penta2*	1102.06	1164.10	0.64	No	564.03	926.54	0.00	Yes	337.43	353.79	0.46	No	1791.02	2108.76	0.00	Yes
*Penta3*	1085.27	1152.15	0.61	No	517.41	867.27	0.00	Yes	330.33	345.89	0.47	No	1696.94	1973.71	0.00	Yes
*PCV1*	1136.80	1126.95	0.94	No	650.72	1060.76	0.00	Yes	333.61	387.24	0.05	No	1876.69	2260.71	0.00	Yes
*PCV2*	1093.94	1164.45	0.59	No	552.61	926.40	0.00	Yes	306.46	356.73	0.03	Yes	1780.71	2107.51	0.00	Yes
*PCV3*	1064.91	1152.05	0.50	No	499.04	859.44	0.00	Yes	298.69	348.73	0.02	Yes	1667.93	2005.17	0.00	Yes
*Rota1*	1008.36	1126.80	0.37	No	548.09	1055.75	0.00	Yes	287.38	386.74	0.00	Yes	1809.21	2253.61	0.00	Yes
*Rota2*	973.34	1164.25	0.16	No	470.15	923.70	0.00	Yes	257.90	353.56	0.00	Yes	1602.88	2105.03	0.00	Yes
*IPV*	1015.08	1151.65	0.30	No	516.64	875.07	0.00	Yes	254.54	367.52	0.00	Yes	1740.37	2074.39	0.00	Yes
*Measles1*	1080.23	1147.85	0.61	No	482.90	853.33	0.00	Yes	360.86	365.79	0.85	No	1663.89	1963.34	0.00	Yes
*Measles2*	990.80	1117.75	0.30	No	249.06	522.80	0.00	Yes	280.59	306.84	0.28	No	1463.89	1616.86	0.02	Yes
	Punjab				Khyber Pakhtunkhwa				Khyber Pakhtunkhwa Tribal Districts				Sindh			
*BCG*	7929.46	10,810.97	0.00	Yes	2901.24	3253.84	0.00	Yes	1089.73	1558.62	0.00	Yes	4676.08	6042.25	0.00	Yes
*OPV0*	7039.70	10,791.53	0.00	Yes	2359.29	2737.75	0.00	Yes	736.74	1051.44	0.00	Yes	3242.00	4348.15	0.00	Yes
*OPV1*	7419.59	9747.04	0.00	Yes	2843.77	3280.18	0.00	Yes	1093.64	1693.27	0.00	Yes	4478.89	5640.20	0.00	Yes
*OPV2*	7123.25	9366.82	0.00	Yes	2601.68	3094.06	0.00	Yes	928.89	1480.97	0.00	Yes	4065.42	5176.58	0.00	Yes
*OPV3*	7057.07	9349.17	0.00	Yes	2501.07	3077.74	0.00	Yes	843.78	1356.41	0.00	Yes	3918.71	5031.80	0.00	Yes
*Penta1*	7316.93	9747.60	0.00	Yes	2847.00	3288.82	0.00	Yes	1089.11	1698.10	0.00	Yes	4479.21	5642.61	0.00	Yes
*Penta2*	7022.49	9377.84	0.00	Yes	2604.59	3097.69	0.00	Yes	930.56	1477.33	0.00	Yes	4065.53	5184.36	0.00	Yes
*Penta3*	6963.90	9366.27	0.00	Yes	2501.44	3085.25	0.00	Yes	845.12	1375.86	0.00	Yes	3917.90	5032.97	0.00	Yes
*PCV1*	7508.54	9718.41	0.00	Yes	2836.73	3280.99	0.00	Yes	1175.14	1695.67	0.00	Yes	4334.81	5628.95	0.00	Yes
*PCV2*	7239.64	9338.28	0.00	Yes	2560.60	3093.35	0.00	Yes	988.22	1481.46	0.00	Yes	3914.67	5165.65	0.00	Yes
*PCV3*	7149.06	9320.20	0.00	Yes	2456.55	3075.35	0.00	Yes	900.70	1379.49	0.00	Yes	3750.86	5033.22	0.00	Yes
*Rota1*	7583.83	9414.10	0.00	Yes	2757.15	3208.62	0.00	Yes	1325.46	1651.67	0.01	Yes	4390.43	5746.51	0.00	Yes
*Rota2*	7237.00	9216.98	0.00	Yes	2383.14	3033.49	0.00	Yes	1114.56	1453.61	0.00	Yes	3842.86	5297.73	0.00	Yes
*IPV*	6969.57	9728.33	0.00	Yes	2482.16	3144.14	0.00	Yes	945.74	1423.68	0.00	Yes	3435.78	5114.83	0.00	Yes
*Measles1*	7192.10	9509.40	0.00	Yes	2369.62	3078.23	0.00	Yes	895.73	1497.60	0.00	Yes	4057.17	5382.15	0.00	Yes
*Measles2*	6264.86	9086.84	0.00	Yes	1657.65	2525.70	0.00	Yes	496.10	1051.98	0.00	Yes	3024.32	4578.91	0.00	Yes

**Table 4:** Number and Percentage of Districts with significant increase in the number of vaccinations.

Province	Districts	BCG	OPV0	OPV1	OPV2	OPV3	Penta1	Penta2	Penta3
AJK	10	0	1	0	1	1	0	1	1
Balochistan	33	27	31	30	30	29	30	30	29
GB	10	3	7	4	4	5	4	4	4
Islamabad	2	2	2	1	1	1	1	1	1
KP	26	17	18	16	19	21	17	18	20
KPTD	7	4	4	5	6	7	7	7	6
Punjab	36	29	32	24	22	23	25	23	26
Sindh	24	22	23	20	21	21	21	21	21
Grand Total	148	104	118	100	104	108	105	105	108
Province	Districts	PCV1	PCV2	PCV3	Rota1	Rota2	IPV	Measles1	Measles2
AJK	10	0	1	1		3	2	0	0
Balochistan	33	31	31	31	33	33	30	32	28
GB	10	4	5	5	6	7	7	3	4
Islamabad	2	2	1	1	2	1	2	1	1
KP	26	17	22	21	18	21	22	20	24
KPTD	7	7	6	6	3	4	6	5	6
Punjab	36	19	20	21	1	6	30	23	31
Sindh	24	20	22	21	19	20	24	22	23
Grand Total	148	100	108	107	82	95	123	106	117
Province	Districts	BCG	OPV0	OPV1	OPV2	OPV3	Penta1	Penta2	Penta3
AJK	10	0%	10%	0%	10%	10%	0%	10%	10%
Balochistan	33	82%	94%	91%	91%	88%	91%	91%	88%
GB	10	30%	70%	40%	40%	50%	40%	40%	40%
Islamabad	2	100%	100%	50%	50%	50%	50%	50%	50%
KP	26	65%	69%	62%	73%	81%	65%	69%	77%
KPTD	7	57%	57%	71%	86%	100%	100%	100%	86%
Punjab	36	81%	89%	67%	61%	64%	69%	64%	72%
Sindh	24	92%	96%	83%	88%	88%	88%	88%	88%
Grand Total	148	70%	80%	68%	70%	73%	71%	71%	73%
Province	Districts	PCV1	PCV2	PCV3	Rota1	Rota2	IPV	Measles1	Measles2
AJK	10	0%	10%	10%	0%	30%	20%	0%	0%
Balochistan	33	94%	94%	94%	100%	100%	91%	97%	85%
GB	10	40%	50%	50%	60%	70%	70%	30%	40%
Islamabad	2	100%	50%	50%	100%	50%	100%	50%	50%
KP	26	65%	85%	81%	69%	81%	85%	77%	92%
KPTD	7	100%	86%	86%	43%	57%	86%	71%	86%
Punjab	36	53%	56%	58%	3%	17%	83%	64%	86%
Sindh	24	83%	92%	88%	79%	83%	100%	92%	96%
Grand Total	148	68%	73%	72%	55%	64%	83%	72%	79%

## Data Availability

The data will be available from the corresponding author upon reasonable request.
